# The LAI–Oral Antipsychotic Efficacy–Effectiveness Paradox in Schizophrenia

**DOI:** 10.3390/medicina62101960

**Published:** 2026-10-10

**Authors:** Kaloyan Stoychev, Sara Begum, Sheraz Nazar

**Affiliations:** 1 Research Institute, Department of Psychiatry and Medical Psychology, Medical University Pleven, 5800 Pleven, Bulgaria; 2 Department of Psychiatry and Medical Psychology, Medical University Pleven, 5800 Pleven, Bulgaria; 1210014@mu-pleven.bg

**Keywords:** schizophrenia, long-acting injectable antipsychotics, oral antipsychotics, medication adherence, treatment adherence, confounding by indication, pharmacogenomics, CYP2D6, CYP3A4, digital medicine, ingestible sensor, pragmatic trials, precision psychiatry, relapse prevention, comparative effectiveness, neuroimaging, positron emission tomography, functional magnetic resonance imaging, artificial intelligence, machine learning, prediction of therapeutic response

## Abstract

*Background and Objectives*: Long-acting injectable (LAI) antipsychotics are associated with reduced hospitalization and relapse in real-world observational studies of schizophrenia; however, randomized controlled trials (RCTs) demonstrate no statistically significant advantage over oral antipsychotics for relapse prevention. This efficacy–effectiveness paradox has remained unresolved in the literature for decades and has directly contributed to the persistent underutilization of LAIs in clinical practice. *Objective*: To examine the methodological sources of the LAI efficacy–effectiveness paradox and to propose four conceptual, precision-medicine-informed methodological solutions, including artificial intelligence (AI)-supported neuroimaging, to guide the design of future comparative effectiveness research. *Materials and Methods*: This report comprises two components: Part I, a scoping review of existing comparative studies, conducted and reported in accordance with the PRISMA extension for scoping reviews (PRISMA-ScR); and Part II, a conceptual methodological framework proposed by the authors on the basis of gaps identified in Part I, which is not itself a product of systematic scoping-review methodology. For Part I, we searched PubMed, EMBASE, PsycINFO, and the Cochrane Library from January 2000 to March 2026. Search terms included ‘long-acting injectable antipsychotics,’ ‘oral antipsychotics,’ ‘schizophrenia,’ ‘medication adherence,’ ‘confounding by indication,’ ‘pharmacogenomics,’ ‘CYP2D6,’ ‘digital medicine,’ ‘pragmatic trials,’ ‘positron emission tomography,’ ‘functional magnetic resonance imaging,’ ‘neuroimaging,’ ‘machine learning,’ and ‘artificial intelligence. *Results:* The paradox is primarily explained by confounding by indication in observational studies and inadequate adherence measurement across all study designs (Part I). On this basis, we propose, as a conceptual framework rather than a validated finding, four methodological solutions (Part II): (1) objective digital adherence monitoring via ingestible sensor or equivalent technology, subject to explicit informed consent and comprehensive ethical safeguards; (2) pharmacogenomic stratification using CYP2D6, CYP3A4, and CYP2C19 metaboliser phenotyping to control for pharmacokinetic confounding specific to antipsychotic drug metabolism; (3) pragmatic RCT designs with real-world eligibility criteria and objective primary adherence endpoints; and (4) artificial intelligence-supported neuroimaging positron emission tomography (PET)-derived dopamine receptor occupancy and machine learning analysis of functional magnetic resonance imaging (fMRI) connectivity data as an objective pharmacodynamic and predictive complement to the three adherence- and pharmacokinetics-focused solutions. *Conclusions:* Current evidence on LAI versus oral antipsychotic superiority cannot be considered definitive. Integration of digital monitoring, pharmacogenomics, pragmatic trial design, and AI-supported PET and fMRI neuroimaging into future research represents a necessary methodological correction that could resolve decades of contradictory findings and provide clinicians with genuinely actionable, patient-centered evidence.

## 1. Introduction

Schizophrenia is a severe, chronic illness affecting approximately 0.3% of the global population, with more than 20 million individuals diagnosed in 2021 [1,2]. It typically begins in late adolescence or early adulthood, profoundly impairs social and occupational functioning, and is associated with substantial premature mortality, reducing life expectancy by an average of 14.5 years [2,3]. Despite the availability of effective antipsychotic medications, outcomes remain suboptimal, driven largely by medication non-adherence, estimated to affect 40% to 75% of patients and to substantially increase the risk of relapse, hospitalization, and functional decline [4,5].

Long-acting injectable (LAI) antipsychotics address non-adherence directly: administered via observed injection every 2 to 12 weeks, they remove the cognitive and organizational burden of daily pill-taking [6]. Large real-world and registry-based studies consistently associate LAI use with reduced hospitalization, lower relapse, and better treatment retention than oral antipsychotics [7,8,9], and clinical practice guidelines, including the American Psychiatric Association’s, recommend LAIs for patients with a history of non-adherence or relapse [6].

This real-world advantage, however, is not reproduced in randomized controlled trials (RCTs), the gold standard for causal inference. Meta-analyses of RCTs comparing LAI and oral antipsychotics have consistently found no significant difference in relapse prevention (Kishimoto et al.: relative risk 0.93, 95% CI 0.80–1.08, across 21 RCTs [10]; Schneider-Thoma et al.: 127 RCTs [11]). This contradiction, which we term the efficacy–effectiveness paradox, suggests that the apparent real-world superiority of LAIs may reflect systematic methodological confounding rather than genuine pharmacological advantage [12,13].

Two opposing biases likely contribute to this paradox: confounding by indication and channeling bias. Confounding by indication, in which clinicians with a preference prescribe LAIs to patients with poor adherence history and higher relapse risk, would if anything be expected to bias observational comparisons against LAIs [12]. We argue that the dominant driver of the observed real-world advantage is instead channeling bias, combined with differential clinical monitoring: patients selected for LAI treatment typically receive more frequent clinician contact and closer relapse monitoring, independent of the depot formulation’s pharmacology [13]. This interpretation is consistent with RCT evidence, in which randomization controls for both patient selection and where assessment is blinded leads to much of the differential monitoring effect.

Aim: This paper aims to (1) synthesize, in accordance with PRISMA-ScR, the evidence underlying the LAI efficacy–effectiveness paradox and its methodological sources (Part I); and (2) propose a conceptual, precision-medicine-informed methodological framework integrating objective adherence monitoring, pharmacogenomic stratification, pragmatic trial design, and AI-supported neuroimaging to guide the design of a definitive future comparative-effectiveness trial (Part II).

Section 3 and Section 4 (Part I) synthesize the existing evidence base and identify four methodological gaps underlying the paradox: inadequate adherence measurement, uncontrolled pharmacokinetic variability, unrepresentative trial populations, and unexploited neuroimaging biomarkers. Section 5 and Section 6 (Part II) present our proposed framework, addressing each gap in turn.

## 2. Methods

This paper reports a scoping review conducted in accordance with the PRISMA (Preferred Reporting Items for Systematic Reviews and Meta-Analyses) extension for scoping reviews. This review was not registered with PROSPERO, as PROSPERO does not currently accept registrations for scoping reviews; no other public registry was used. We searched PubMed, EMBASE, PsycINFO, and the Cochrane Library from January 2000 to March 2026. The following MeSH terms and free-text keywords were used in combination: ‘long-acting injectable antipsychotics,’ ‘depot antipsychotics,’ ‘oral antipsychotics,’ ‘schizophrenia,’ ‘schizoaffective disorder,’ ‘medication adherence,’ ‘treatment adherence,’ ‘confounding by indication,’ ‘selection bias,’ ‘pharmacogenomics,’ ‘CYP2D6,’ ‘CYP3A4,’ ‘CYP2C19,’ ‘cytochrome P450,’ ‘digital medicine,’ ‘ingestible sensor,’ ‘pragmatic trial,’ ‘PRECIS-2,’ ‘positron emission tomography,’ ‘dopamine receptor occupancy,’ ‘functional magnetic resonance imaging,’ ‘resting-state connectivity,’ ‘machine learning,’ ‘artificial intelligence,’ ‘neuroimaging biomarker,’ and ‘comparative effectiveness.’ No language restrictions were applied during the initial search.

Studies were included if they: (1) reported comparative effectiveness or adherence data for LAI versus oral antipsychotics in adult populations diagnosed with schizophrenia or schizoaffective disorder; (2) were primary research studies (RCTs, cohort studies, mirror-image studies, or cross-sectional surveys), systematic reviews, or meta-analyses; or (3) addressed methodological issues relevant to LAI versus oral antipsychotic research, including confounding by indication, adherence measurement approaches, CYP450 pharmacogenomics in antipsychotic prescribing, pragmatic trial design methodology, or the application of PET, fMRI, or artificial intelligence/machine learning methods to antipsychotic pharmacodynamics or treatment-response prediction. Case reports, conference abstracts without full data, and editorials were excluded. Reference lists of included systematic reviews were additionally searched for relevant primary studies not identified through database searching. Titles and abstracts were screened independently by two reviewers, with disagreements resolved through discussion and, where necessary, arbitration by a third reviewer.

The methodological quality of included primary studies was assessed using validated tools: the Cochrane Risk of Bias tool version 2 (RoB 2) for RCTs, the Risk of Bias in Non-randomized Studies of Interventions (ROBINS-I) tool for observational studies, and the AMSTAR-2 (A Measurement Tool to Assess systematic Reviews, version 2) for included systematic reviews and meta-analyses.

Quality assessment findings for the primary studies informing this review are summarized in Supplementary Table S1 submitted alongside this manuscript. Quality ratings across the ten primary studies ranged from low to high, with two rated high, one moderate to high, four moderate, two low to moderate, and one low. No study was judged critically flawed, with residual confounding by indication and heterogeneity in follow-up duration being the main concerns.

A summary of the evidence comparing LAI and oral antipsychotics across study designs, including their main limitations and the corresponding proposed solutions, is presented in Table 1. Additionally, Table 2 summarizes the number and type of studies contributing evidence within each domain.

### Study Selection and Characteristics

**Figure 1:**
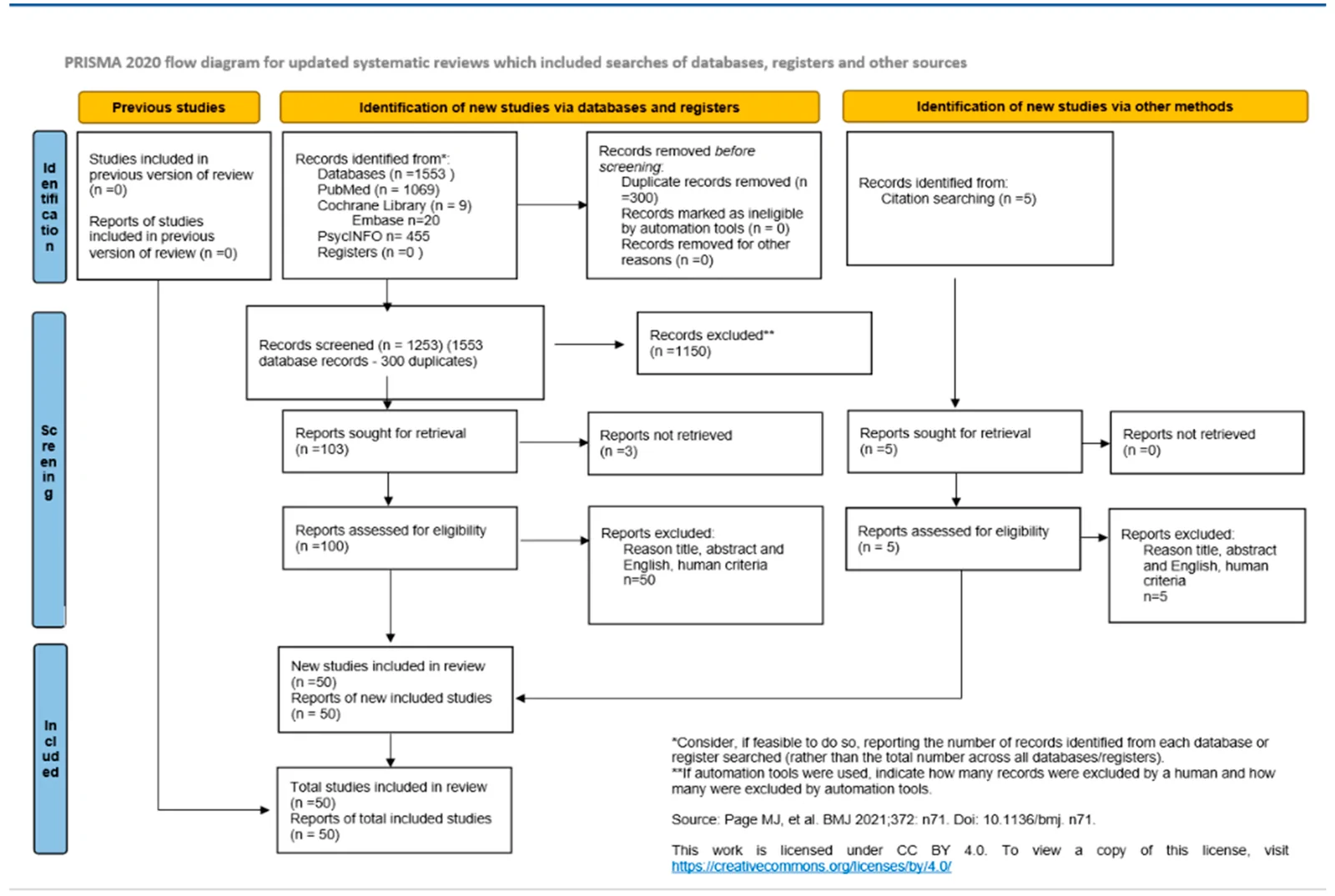
PRISMA 2020 flow diagram for updated systematic reviews which included searches of databases, registers and other source: [14]. **Note**—A total of 1553 records were identified across four electronic databases (PubMed: n = 1069; EMBASE: n = 20; PsycINFO: n = 455; Cochrane Library: n = 9). After removing 300 duplicates, 1253 records underwent title and abstract screening, resulting in 1150 exclusions. Of the 103 full-text reports sought, 3 were unretrievable. Eligibility assessment of the remaining 100 full-text articles led to 50 exclusions. A final total of 50 studies met all criteria and were included in the review synthesis.

Study selection followed the process summarized in Figure 1 (PRISMA-ScR flow diagram). Database searching identified 1553 records (PubMed, n = 1069; EMBASE, n = 20; PsycINFO, n = 455; Cochrane Library, n = 9). After removal of 300 duplicate records, 1253 records underwent title and abstract screening, of which 1150 were excluded. A total of 103 reports were sought for retrieval, three reports could not be retrieved, and 100 full text reports were assessed for eligibility. Following full text assessment, 50 studies met the inclusion criteria and were included in the scoping review, Figure 1.

**Table 1:** Summary of evidence on LAI versus oral antipsychotics by study design, key findings, limitations, and methodological gaps addressed by the four proposed solutions.

Study Design	Key Examples	Primary Finding	Main Limitation	Addressed by Proposed Solution
Explanatory RCTs	Kishimoto et al. 2014 [10]; Schneider-Thoma et al. 2022 [11]	No significant difference in relapse rates (RR 0.93, 95% CI 0.80–1.08). LAI and oral antipsychotics are statistically equivalent.	Unrepresentative, highly selected populations; compresses adherence advantage; no objective adherence measurement.	Pragmatic RCT design (solution 3); digital adherence monitoring (solution 1).
Real-World Cohort Studies	]; Lin et al. 2021 [7]; Tiihonen et al. 2011 [8]; Tiihonen et al. 2017 [9]; Efthimiou et al. 2024 [15]	LAI use associated with lower mortality, fewer hospitalisations, and better treatment persistence.	Confounding by indication; proxy adherence measurement; no pharmacogenomic control; monitoring intensity as co-intervention.	Digital adherence monitoring (solution 1); pharmacogenomic stratification (solution 2); pragmatic RCT design (solution 3).
Mirror-Image Studies	Kishimoto et al. 2013 [16]	LAI associated with risk ratio of 0.43 for hospitalization (95% CI 0.35–0.53) compared with prior oral treatment periods.	Post confounding; regression to the mean; no objective adherence measurement in oral period.	Partially addressed by pragmatic RCT design (solution 3) and digital monitoring (solution 1) in future designs.
Pragmatic RCTs	Kane et al. 2020 PRELAPSE [17]	Encouraging LAI use significantly delayed time to first hospitalization in early-phase schizophrenia (HR 0.56, 95% CI 0.34–0.92).	Cluster randomisation limits individual-level causal inference; no pharmacogenomic stratification; adherence by injection records only.	Pharmacogenomic stratification (solution 2); digital monitoring in oral arms (solution 1); expanded eligibility.
PET/fMRI and AI-Based Neuroimaging Studies	Uchida & Suzuki 2014 [18]; Mehta et al. 2021 [19]; Cao et al. 2023 [20]	High, sustained D2/D3 receptor occupancy with LAIs (PET); ML-derived fMRI connectivity predicts antipsychotic response with 81% sensitivity/76% specificity.	Occupancy data derived almost exclusively from oral formulations; no LAI-versus-oral head-to-head occupancy-stability study; fMRI prediction models not yet validated in LAI eligible populations or tested against adherence-confirmed oral arms.	AI-supported neuroimaging (solution 4); complements pragmatic RCT design (solution 3) and pharmacogenomic stratification (solution 2).

AI, artificial intelligence; CI, confidence interval; fMRI, functional magnetic resonance imaging; HR, hazard ratio; LAI, long-acting injectable antipsychotic; ML, machine learning; PET, positron emission tomography; RCT, randomized controlled trial; RR, risk ratio.

Table 2 summarizes the number and type of studies contributing evidence within each domain addressed by this review, distinguishing empirical comparative-effectiveness studies from methodological guidance sources and from the pharmacogenomic, PET, fMRI/machine-learning, and digital-monitoring studies that inform the proposed framework in Part II.

**Table 2:** Number and type of studies contributing evidence, by domain.

Evidence Domain	N Studies/Sources	Representative References
Explanatory RCTs/RCT meta-analyses (comparative effectiveness)	2	Kishimoto et al. 2014 [10] (21 RCTs); Schneider-Thoma et al. 2022 [11] (127 RCTs)
Real-world cohort/registry studies (comparative effectiveness)	5	Tiihonen et al. 2011 [8]; Tiihonen et al. 2017 [9]; Lin et al. 2021 [7]; Taipale et al. 2022 [21]; Efthimiou et al. 2024 [15]
Mirror-image studies (comparative effectiveness)	1	Kishimoto et al. 2013 [16]
Pragmatic RCTs (comparative effectiveness)	1	Kane et al. 2020 PRELAPSE [17]
Methodological/trial-design guidance (non-empirical)	2	Kyriacou & Lewis 2016 [12]; Loudon et al. 2015 PRECIS-2 [22]
Pharmacogenomic/CYP450 pharmacokinetic studies and guidelines	5	Khani et al. 2024 [23]; Nip et al. 2021 [24]; Swen et al. 2023 [25]; Belmonte et al. 2018 [26]; Swen et al. 2011 DPWG [27]
PET receptor-occupancy studies	3	Uchida & Suzuki 2014 [18]; Gefvert et al. 2005 [28]; Schmitz et al. 2024 [29]
fMRI/machine-learning response-prediction studies	5	Mehta et al. 2021 [19]; Cao et al. 2023 [20]; Cui et al. 2020 [30]; Ambrosen et al. 2020 [31];
Digital adherence monitoring studies	2	Thorpe et al. 2009 [32]; Wu et al. 2023 [33]

Formal quality/risk-of-bias assessment (Supplementary Table S1) was completed for the ten primary comparative-effectiveness and methodological studies most central to the paradox addressed in this review, using Rob 2 for randomized trials, ROBINS-I for observational studies, and AMSTAR-2 for systematic reviews and meta-analyses.

PART I: SCOPING REVIEW OF THE EVIDENCE

Section 3 and Section 4 report findings from the systematic search and evidence appraisal described in Part I of the Methods (Section 2). No forward-looking or conceptual proposals are presented in this Part.

## 3. Background

### 3.1. Schizophrenia and the Non-Adherence Crisis

Medication non-adherence in schizophrenia is a multifactorial problem driven by a complex interaction of patient-level, treatment-level, and system-level factors [4,5]. At the patient level, lack of illness insight, known clinically as anosognosia, is present in approximately 50% of patients with schizophrenia and represents the single strongest predictor of non-adherence, as patients who do not recognize their illness have little motivation to take medication consistently [34]. Negative beliefs about medication, including fears regarding dependency, side effects, and loss of personal identity, further undermine treatment engagement [4]. Co-occurring substance use disorders, which are present in up to 50% of individuals with schizophrenia, induce adherence difficulties by disrupting routine, impairing executive function, and creating competing behavioral priorities [35].

At the treatment level, the burden of daily oral medication, particularly when associated with significant adverse effects such as weight gain, metabolic disturbance, sedation, sexual dysfunction, and extrapyramidal symptoms, makes sustained adherence cognitively and organizationally demanding for patients who already experience executive function deficits [4,36].

The consequences of medication non-adherence in schizophrenia are severe, progressive, and extend well beyond symptom recurrence. Non-adherence is the strongest modifiable predictor of relapse, accounting for an estimated 40% to 50% of acute psychotic episodes [30,37].

At the system level, fragmented care, poor continuity between inpatient and community services, inadequate therapeutic relationship quality between patient and prescriber, and economic barriers collectively create an environment in which non-adherence becomes the path of least resistance [4,5].

In turn, each relapse carries a three- to fivefold increase in the risk of psychiatric hospitalization [22,38]. Repeated relapses drive a pattern of progressive functional deterioration, occupational, social, and interpersonal, alongside a deepening negative attitude toward medication and treatment engagement [4]. Furthermore, the recurrent relapse-associated exacerbation of distressing positive symptoms accompanied by feelings of hopelessness and psychosocial devastation mediate the high suicide risk in patients with schizophrenia from which about 10% die by suicide [36,39]. The economic burden is equally significant: for example, the total cost of schizophrenia in the United States in 2024 was estimated at $366.8 billion, with indirect costs including lost productivity, premature mortality and unpaid caregiving accounting for the major share of it [40,41].

A critical and often underappreciated feature of schizophrenia is that relapse is not simply a return to a prior clinical baseline. Each episode causes lasting neurobiological damage that worsens long-term prognosis [38,39]. This includes progressive grey matter volume loss, particularly in frontal and temporal regions, and increasing dopaminergic dysregulation that lowers the threshold for future psychotic decompensation [39]. This sensitization process explains why early, sustained adherence is not merely clinically beneficial but neurobiologically critical: each successive episode produces more severe symptoms, greater treatment resistance, and a longer recovery trajectory [38]. Patients who experience multiple relapses demonstrate a rising probability of transitioning to treatment-resistant schizophrenia, for which even optimal pharmacotherapy, including clozapine, produces limited benefit [42].

### 3.2. Long-Acting Injectables: Pharmacology and Clinical Rationale

Long-acting injectable antipsychotics were developed to overcome the pharmacokinetic limitations of oral formulations and the behavioral challenge of daily self-administration. LAIs deliver antipsychotic medication in oil-based or aqueous microsphere depot formulations administered intramuscularly or subcutaneously, producing slow, sustained drug release that maintains stable plasma concentrations over weeks to months [6,43,44].

This pharmacokinetic profile eliminates the peak-to-trough plasma concentration fluctuations associated with daily oral dosing, which are implicated in breakthrough symptoms and side-effect variability. First-generation LAIs, including haloperidol decanoate and fluphenazine decanoate, available since the 1960s and 1970s, demonstrated clinical efficacy but carried significant extrapyramidal side-effect burdens that limited acceptability. Second-generation LAIs, including risperidone microspheres, paliperidone palmitate (available in once-monthly and once-quarterly formulations), aripiprazole monohydrate, aripiprazole lauroxil, and olanzapine pamoate, offer improved tolerability profiles alongside extended injection intervals ranging from two weeks to three months [6,45].

Beyond the pharmacokinetic advantage, LAIs offer a diagnostically important clinical benefit that is rarely discussed in the comparative effectiveness literature. When a patient receiving an LAI experiences a relapse, the treating clinician can be confident that medication delivery occurred as scheduled, immediately shifting the diagnostic focus toward pharmacodynamic factors, dose adequacy, emergent treatment resistance, or psychosocial precipitants, rather than adherence uncertainty [13]. Despite a compelling theoretical rationale and a growing body of real-world evidence, LAIs remain substantially underutilized in clinical practice.

They are estimated to be prescribed to only 20% to 30% of patients who might clinically benefit, and in early-phase schizophrenia, LAI use has been reported as low as 4% in a commercially insured United States population [6,42,46]. Persistent negative perceptions rooted in first-generation LAI experience, prescriber reluctance, patient stigma regarding injections, and healthcare system barriers collectively contribute to this implementation gap.

### 3.3. The Evidence Base: What We Know and What We Do Not

#### 3.3.1. RCT Evidence

Randomized controlled trials, which provide the strongest causal evidence, consistently show no significant difference between LAI and oral antipsychotics for relapse prevention. Kishimoto et al. conducted a meta-analysis of 21 RCTs and found a relative risk of 0.93 (95% CI 0.80–1.08), statistically equivalent outcomes [10]. A subsequent network meta-analysis by Schneider-Thoma et al. of 127 RCTs confirmed this finding across multiple LAI and oral formulations [11]. Investigators have attributed the null findings of explanatory RCTs, in part, to systematic differences between trial and community populations: participants in RCTs are typically more adherent, less severely ill, and more closely monitored than average clinical patients, thereby compressing the adherence advantage that LAIs would be expected to confer [47].

#### 3.3.2. Real-World and Mirror-Image Evidence

In contrast, real-world observational studies and mirror-image analyses consistently favor LAIs. Mirror-image studies, which compare pre-LAI versus post-LAI periods within the same patient, show particularly strong effects, with LAIs associated with a risk ratio of 0.43 for hospitalization (95% CI 0.35–0.53) compared with prior oral treatment periods [16]. Cohort studies from national registries, including the Finnish dataset analyses by Tiihonen et al. (2011) and the Swedish cohort reported by Tiihonen et al. (2017), demonstrate that LAI use is associated with 20% to 33% lower mortality and substantially reduced rehospitalization compared with oral antipsychotics over multi-year follow-up [8,9]. A landmark combined analysis by Efthimiou et al. (2024) found that the advantage of LAIs over oral antipsychotics is systematically larger in real-world settings than in RCT settings, confirming the scale of the efficacy–effectiveness gap [15].

#### 3.3.3. First-Episode Versus Multi-Episode Schizophrenia: A Distinct Evidence Gap

The evidence base for LAIs differs substantially between first-episode and multi-episode schizophrenia populations, and this distinction has not been adequately addressed in existing comparative effectiveness reviews. The landmark PRELAPSE trial by Kane et al., a cluster-randomized pragmatic trial specifically in early-phase schizophrenia, demonstrated that encouraging LAI use significantly delayed time to first hospitalization (hazard ratio 0.56, 95% CI 0.34–0.92) [17]. However, the biological and psychosocial context of first-episode psychosis differs markedly from multi-episode illness: insight deficits may be less entrenched, attitudes toward medication are less consolidated, and neurobiological sensitization is less advanced. LAI acceptability and the magnitude of the adherence advantage may therefore differ systematically between these populations. Future comparative effectiveness research should stratify by illness phase at enrolment and report outcomes separately for first-episode and multi-episode participants.

## 4. Evidence Gaps and Methodological Limitations

### 4.1. Confounding by Indication and Channeling Bias

Confounding by indication and channeling bias are distinct methodological threats to causal inference in observational LAI versus oral antipsychotic studies, and they are frequently conflated in the literature. As formally defined by Kyriacou and Lewis, confounding by indication occurs when the clinical reason for prescribing a treatment is itself an independent predictor of the outcome being measured [12]. In the LAI context, clinicians preferentially prescribe LAIs to patients with poor adherence histories, high relapse risk, and greater illness severity, precisely the patient characteristics that independently predict worse clinical outcomes. Applied consistently, this mechanism would be expected to bias observational comparisons against LAIs: if LAI recipients are systematically harder to treat at baseline, unadjusted or incompletely adjusted, comparisons should, if anything, understate any true benefit of LAIs relative to oral antipsychotics.

The apparent real-world superiority of LAIs is therefore better explained by a second, distinct mechanism: channeling bias combined with differential clinical monitoring. Patients selected for LAI treatment, and the structured clinical pathways through which LAIs are typically prescribed and administered, often involve more frequent clinician contact, closer monitoring for early signs of relapse, and greater engagement with psychosocial support, independent of the pharmacological properties of the depot formulation itself [13]. Because more intensive monitoring and support plausibly reduce relapse risk directly, this mechanism operates in the opposite direction to confounding by indication and can more parsimoniously account for the observed real-world LAI advantage.

Attempts to control for these biases using propensity score matching and multivariate regression analysis have provided partial mitigation but cannot fully resolve the problem when key confounders, including real-time adherence behavior, therapeutic alliance quality, monitoring intensity, and degree of illness insight, are not directly measured [48]. The efficacy–effectiveness paradox may therefore reflect the net result of two biases operating in opposite directions: confounding by indication, which works against LAIs, and channeling bias/differential monitoring, which works in their favor and appears to dominate in the observational literature. This interpretation is consistent with RCT evidence, in which randomization controls for patient selection and, where outcome assessment is blinded, substantially reduces differential monitoring effects, and in which LAI and oral antipsychotics show statistical equivalence for relapse prevention [10,11].

### 4.2. Adherence Measurement Failures

A foundational problem across the entire LAI versus oral comparative effectiveness literature is the near-universal absence of objective adherence measurement. The overwhelming majority of studies, including those incorporated into the Lin et al. and Kishimoto et al. meta-analyses, rely on pharmacy refill records, medication possession ratios, or self-report to estimate oral antipsychotic adherence [7,10]. These measures systematically overestimate true adherence: a patient who collects a prescription is recorded as adherent even if the medication is not subsequently ingested.

Self-report is further subject to social desirability bias, particularly in patients with limited illness insight who may not accurately perceive their own non-adherence [34]. The consequence of this measurement asymmetry is that studies comparing LAI (where adherence is guaranteed by clinician-administered injection) to oral antipsychotics (where adherence is inferred from proxy measures) are not comparing equivalent quantities. The apparent adherence advantage of LAIs in such studies may be partly or entirely artefactual, a product of measurement asymmetry rather than pharmacological difference [13].

### 4.3. Population Generalizability

The populations enrolled in LAI versus oral RCTs are systematically unrepresentative of the real-world schizophrenia population in ways that directly undermine the validity of comparative effectiveness conclusions. Standard RCT exclusion criteria eliminate patients with active substance use disorders, significant medical comorbidities, current suicidality, forensic history, and treatment resistance, collectively representing a substantial proportion of patients encountered in community mental health settings [10,47]. A landmark cohort study by Efthimiou et al. 2024 [15] applied eligibility criteria from 53 typical schizophrenia RCTs to national patient registries in Finland and Sweden, finding that approximately 79% of real-world individuals with schizophrenia spectrum disorders would be ineligible for inclusion [16]. Critically, these ineligible patients demonstrated substantially worse outcomes, including higher hospitalization rates, than those who would qualify for trial participation [21] confirming that the populations generating current comparative effectiveness evidence are systematically less ill than those for whom clinicians most need guidance.

### 4.4. Pharmacokinetic Variability and Pharmacogenomic Confounding

Existing LAI versus oral antipsychotic studies have largely neglected the role of pharmacogenomic variability in confounding comparative effectiveness findings. Individual differences in cytochrome P450 enzyme activity, determined by genetic variants in CYP2D6, CYP3A4, and CYP2C19, produce substantial variability in antipsychotic plasma concentrations at standard oral doses, particularly for risperidone, aripiprazole, and their active metabolites [23,24,49].

A systematic review and meta-analysis specifically examining CYP2D6 genetic polymorphisms and risperidone pharmacokinetics confirmed that CYP2D6 phenotype significantly affects plasma concentrations and is associated with differential adverse effect burden, which may in turn trigger non-adherence [24]. A patient classified as a CYP2D6 poor metabolizer receiving a standard oral risperidone dose may experience drug exposure equivalent to two to three times that of an extensive metabolizer, resulting in adverse effects, particularly weight gain and extrapyramidal symptoms, that drive intentional non-adherence [24]. Conversely, an ultra-rapid metabolizer on the same dose may achieve subtherapeutic plasma concentrations despite nominal dosing compliance, appearing treatment-resistant when the problem is pharmacokinetic rather than pharmacodynamic [23].

It is important to characterize precisely which sources of pharmacokinetic variability LAI administration does and does not address. Intramuscular or subcutaneous injection bypasses gastrointestinal absorption and first-pass hepatic and intestinal metabolism, removing one specific source of variability that affects oral bioavailability. It does not, however, eliminate CYP-mediated systemic metabolism: once the drug enters the systemic circulation from the injection depot, clearance via CYP2D6, CYP3A4, or CYP2C19 remains genotype-dependent, so CYP450-related variability in steady-state plasma concentration and adverse-effect burden should be expected to persist, at least in part, with LAI formulations [23]. Whether the depot’s slow-release pharmacokinetics meaningfully attenuate the clinical consequences of this variability relative to oral dosing of the same drug, for example by reducing peak-concentration-driven adverse effects, has not been isolated and quantified in a properly genotype-stratified trial.

This remains a major unaddressed source of confounding in the comparative effectiveness literature, and represents a specific, testable pharmacogenomic hypothesis rather than an established pharmacokinetic advantage of the LAI formulation [23,24].

### 4.5. Unexploited Neuroimaging and AI Biomarkers

A final, critical evidence gap in the comparative effectiveness literature is the absence of brain-level pharmacodynamic and predictive biomarkers. Existing LAI versus oral comparative studies have relied almost exclusively on behavioral or administrative proxies of response (e.g., relapse or hospitalization) without incorporating objective measures of dopamine receptor engagement or neural circuit function. Consequently, current evidence cannot establish whether sustained receptor occupancy translates into distinct neurobiological outcomes independent of adherence and monitoring effects.

A complete cross-walk summarizing the evidence gaps identified in Part I alongside their corresponding methodological solutions proposed in Part II is presented in Table 3.

PART II: PROPOSED METHODOLOGICAL FRAMEWORK

Section 5 and Section 6 present the authors’ original conceptual framework, developed in response to the evidence gaps identified in Part I (Section 4). This framework is a forward-looking proposal for future research design; it is not a product of the scoping-review methodology and should not be interpreted as an established or validated finding.

## 5. Proposed Methodological Solutions

### 5.1. Objective Digital Adherence Monitoring

The most direct solution to the adherence measurement problem in oral antipsychotic arms of comparative effectiveness trials is the use of ingestible sensor technology to objectively confirm medication ingestion in real time. In 2017, the United States Food and Drug Administration approved the first digital medicine system for a psychiatric indication, ABILIFY MyCite (aripiprazole with ingestible sensor), which embedded a 1 mm ingestible event marker sensor in an aripiprazole tablet [50]. When the tablet contacted gastric fluid, the sensor activated and transmitted an ingestion signal to a wearable patch, which relayed the data to a smartphone application and secure web portal accessible to the patient, caregiver, and clinician. This system provided objective, timestamped confirmation of ingestion, a qualitative advance over any proxy measure currently used in comparative effectiveness research. It is important to acknowledge, however, that ABILIFY MyCite was subsequently discontinued from commercial distribution by Otsuka Pharmaceutical. The underlying ingestible sensor technology remains an important proof-of-concept for objective adherence monitoring in psychiatric populations, and equivalent digital medicine systems are in active development and clinical validation for psychiatric indications [33]. Evidence for the acceptability and feasibility of digital medicine systems in patients with serious mental illness, including schizophrenia, is accumulating. A two-site, provider-randomized feasibility trial conducted within the Veterans Health Administration demonstrated that a digital medicine system using aripiprazole with ingestible sensor was acceptable to patients with schizophrenia, schizoaffective disorder, bipolar disorder, and major depressive disorder, with a mean proportion of days covered of 0.85 among those in the digital medicine arm [42].

A broader scoping review of digital health interventions to improve adherence to oral antipsychotics among patients with schizophrenia concluded that digital health interventions were acceptable and feasible, with their implementation being possible across diverse settings, although the review identified a lack of fully validated randomized evidence and a need for universal adherence measurement instruments [32,33].

The research methodology proposed here is not dependent on any single commercial platform, but on the broader principle of objectively confirmed ingestion, which is achievable through multiple sensor and digital monitoring approaches currently under evaluation.

In the context of a LAI versus oral antipsychotic comparative effectiveness trial, digital adherence monitoring would serve as the primary objective adherence endpoint for the oral antipsychotic arm, enabling a genuine, symmetrical comparison with the confirmed drug delivery inherent in LAI administration. Studies using this methodology could, for the first time, directly test whether LAI pharmacokinetics confer clinical advantage independent of the adherence guarantee. Furthermore, digital monitoring data would enable a principled distinction between intentional and unintentional non-adherence, permitting subgroup analyses those current studies are unable to perform [33].

### 5.2. Pharmacogenomic Stratification

Pharmacogenomic stratification represents a methodologically robust approach to controlling for pharmacokinetic confounding that has been systematically overlooked in LAI versus oral comparative effectiveness research. By genotyping trial participants for CYP2D6, CYP3A4, and CYP2C19 variants prior to randomization, future trials can stratify participants by metabolizer phenotype and adjust oral antipsychotic doses accordingly, ensuring that oral arm participants achieve therapeutic plasma drug concentrations that are pharmacokinetically equivalent to those achieved in LAI arm participants [23,24].

This design isolates the true effect of the delivery mechanism, injectable versus oral, from the confounding effect of pharmacokinetic variability. A systematic review and meta-analysis by Bousman et al. confirmed that pharmacogenetic-guided antipsychotic prescribing is either superior to or equivalent to standard prescribing for both clinical and economic outcomes across psychiatric populations, providing evidence-based support for the integration of pharmacogenomic testing into trial design [23].

The practical implementation of pharmacogenomic stratification requires a single saliva or blood sample at baseline, which can be processed using commercially available, validated multi-gene pharmacogenomic panels within one to two weeks; a large multicenter implementation trial has confirmed the feasibility of pre-emptive multi-gene panel testing at scale in routine clinical workflows [15]. Dose adjustment guidance for antipsychotics based on CYP2D6 metabolizer status is specifically available through the Dutch Pharmacogenetics Working Group (DPWG), which provides evidence-based dose recommendations for risperidone and aripiprazole according to CYP2D6 phenotype [27]. Importantly, the CPIC guideline cited in earlier versions of this work [37] pertains to serotonin reuptake inhibitors and is not applicable to antipsychotic dosing; the DPWG guidelines for antipsychotics represent the appropriate clinical standard [27]. Incorporation of pharmacogenomic stratification adds a modest estimated cost of $100 to $500 per participant for multi-gene panel testing, which is likely offset by the substantial improvement in causal inference from controlling for a major source of interindividual pharmacokinetic heterogeneity.

### 5.3. Pragmatic Randomized Trial Design

Pragmatic randomized controlled trials, designed to test interventions under conditions closely mirroring real-world clinical practice, represent the optimal study design for resolving the LAI versus oral antipsychotic comparative effectiveness question [22,51]. Unlike explanatory RCTs, which prioritize internal validity through strict eligibility criteria and highly protocol-driven care, pragmatic trials maximize external validity by enrolling representative patient populations, permitting flexibility in co-administered treatments, and measuring outcomes of direct relevance to patients and health systems. The PRECIS-2 framework provides an established, validated tool for positioning trials on the pragmatic-to-explanatory continuum across nine design domains; future LAI comparison trials should explicitly target high pragmatism across all relevant domains and report their PRECIS-2 scores [22]. A pragmatic design for LAI versus oral comparison would incorporate patients with substance use comorbidities, multiple prior relapses, and varying degrees of treatment resistance, precisely the populations that current explanatory trials exclude but that are most likely to benefit from LAI treatment [17]. Real-world claims data further indicate that even partial non-adherence is associated with a higher risk of rehospitalization, which underscores the need for objective adherence measurement within such trials [52].

The integration of the three key trial-design solutions, objective digital adherence monitoring, pharmacogenomic stratification, and pragmatic trial design, into a single study protocol would generate comparative effectiveness evidence of a qualitatively superior order to any currently available study. Such a trial would provide confirmed adherence data in the oral arm, pharmacokinetically calibrated dosing across CYP450 metabolizer strata, a clinically representative enrolled population, and the causal rigors of randomized treatment assignment, simultaneously addressing all four major methodological gaps identified in Section 4.

The resulting data would, for the first time, enable a genuine and definitive test of whether LAI pharmacokinetics confer clinical advantage when adherence and pharmacokinetic variability are held constant. As described in Section 5.4, nesting an AI-supported PET and fMRI imaging sub study within this same trial would add a fourth, brain-based layer of objective pharmacodynamic and predictive evidence that none of the other three solutions can independently provide.

### 5.4. Artificial Intelligence-Supported Neuroimaging: PET and fMRI as Objective Pharmacodynamic and Predictive Markers

A fourth, complementary methodological solution arises from the convergence of molecular and functional neuroimaging with artificial intelligence (AI) and machine learning (ML) analytics. Whereas the three solutions described above address adherence measurement, pharmacokinetic confounding, and trial population representativeness, none directly addresses the brain-level pharmacodynamic question of whether stable, sustained dopaminergic blockade, the pharmacological rationale for LAI formulations, translates into a measurably different neurobiological or functional outcome compared with adherence-confirmed oral treatment. Positron emission tomography (PET) and functional magnetic resonance imaging (fMRI), increasingly analyzed using AI-based quantitative and predictive modeling rather than conventional region-of-interest statistics alone, offer a route to closing this gap.

#### 5.4.1. PET Imaging of Dopamine Receptor Occupancy: An Objective Pharmacodynamic Endpoint

PET imaging using D2/D3 receptor radioligands has been used since the late 1980s to establish that antipsychotic clinical response requires striatal dopamine receptor occupancy of approximately 65% to 80%, with occupancy above 80% associated with a substantially increased risk of extrapyramidal side effects [18]. A systematic review of 20 PET and SPECT studies quantifying D2 receptor occupancy with first- and second-generation LAI antipsychotics, including haloperidol decanoate, risperidone microspheres, and olanzapine pamoate, demonstrated high and sustained receptor blockade over the dosing interval, but also found that the optimal dose and dosing frequency for LAI maintenance treatment remains poorly defined because the great majority of receptor-occupancy data have been derived from oral formulations rather than from LAIs directly [18]. Comparative PET data on risperidone LAI found that occupancy varied considerably with dose (25 mg, 50 mg, and 75 mg injections corresponding to occupancy ranges of approximately 25–48%, 59–83%, and 62–72%, respectively), and indirect comparisons between oral paliperidone extended-release and paliperidone palmitate LAI suggest broadly comparable steady-state striatal occupancy once dose-equivalence is accounted for [18,28]. These findings are directly relevant to the efficacy–effectiveness paradox addressed in this paper. No published PET study has directly compared receptor occupancy stability, the within-patient variance in occupancy across the dosing interval, between an LAI arm and an adherence-confirmed oral arm of the same molecule. Such a study would provide a genuinely objective pharmacodynamic endpoint, independent of self-report or pharmacy-refill proxies, and would directly test whether the smoother pharmacokinetic profile of LAIs translates into more stable receptor engagement and, by extension, more stable clinical response. AI-based quantitative methods, including automated region-of-interest segmentation and kinetic modeling of PET time-activity curves, have begun to reduce inter-rater variability in occupancy estimation and could allow such comparisons to be conducted at a scale and consistency not previously achievable with manual analysis [29].

#### 5.4.2. fMRI Connectivity and Machine Learning Prediction of Antipsychotic Response

A separate and substantial body of evidence has used resting-state functional MRI (rs-fMRI) connectivity, analyzed with machine learning, to predict individual antipsychotic treatment response before treatment is initiated. A systematic review and meta-analysis of 22 datasets comprising 1280 individuals found that striatal and default-mode network connectivity metrics were consistent predictors of subsequent response, with a pooled correlation of approximately 0.47 for continuous symptom improvement and a pooled odds ratio of 12.66 (81% sensitivity, 76% specificity) for categorically defined response [19]. Building on this, Cao et al. developed a functional connectome-based predictive model in first-episode psychosis that generated an individualized, quantitative response score from baseline connectivity data, demonstrating that machine learning models trained on fMRI features can generalize across independent patient cohorts [20]. Complementary radiomics approaches combining structural and functional MRI features have reported prediction accuracies for early treatment response of approximately 80% [30], and multimodal ML frameworks integrating neuroimaging with clinical and neurocognitive features have shown comparable performance using algorithms such as random forests and gradient boosting [31,37]. Critically for the present review, this entire body of ML-based fMRI prediction literature has been generated almost exclusively in patients treated with oral antipsychotics; to our knowledge, no study has yet used connectome-based machine learning models to compare predicted versus observed response between LAI and oral treatment arms, nor to test whether baseline connectivity signatures interact with formulation (LAI versus oral) to predict differential outcome. This represents a clear and specific evidence gap.

If AI-derived connectivity biomarkers prospectively identify which patients are likely to respond poorly to standard oral treatment because of intrinsic network-level vulnerability rather than non-adherence, such biomarkers could be incorporated as a stratification variable at randomization in the pragmatic trial design proposed in Section 5.3, distinguishing, for the first time, patients whose poor outcomes reflect a true pharmacodynamic non-response from those whose poor outcomes reflect unconfirmed non-adherence or pharmacokinetic underdosing.

#### 5.4.3. Methodological Risks of ML-Based Prediction: From Proof-of-Concept to Clinical Utility

The machine learning-based fMRI prediction literature summarized in Section 5.4.2 currently constitutes proof-of-concept evidence, not a validated clinical prediction tool, and this distinction should be maintained explicitly rather than implied. Several methodological risks limit the present translational readiness of this literature and would need to be directly addressed before any LAI-eligible stratification application could be considered clinically actionable.

First, overfitting is a material concern: several of the models reported achieving 75–85% sensitivity/specificity or approximately 80% prediction accuracy were developed and tested in modestly sized single-cohort samples [19,20,21,22,23,24,25,26,27,28,29,30], and performance in such settings is known to be an optimistic estimate of true generalization performance unless rigorously cross-validated and externally tested. Second, dataset shift, systematic differences between the population and acquisition conditions used to train a model and those in which it is subsequently applied, is a specific and unaddressed threat here, because no existing model has been trained or tested in an LAI-eligible population with documented adherence difficulty; applying models trained in adherent, oral-treatment cohorts to this materially different population cannot be assumed to preserve reported accuracy.

Third, scanner and site heterogeneity in fMRI acquisition parameters, hardware, and preprocessing pipelines is a well-documented source of performance degradation in multi-site neuroimaging prediction models and would require harmonized acquisition protocols and, ideally, harmonization methods applied at the analysis stage.

Fourth, few of the cited studies report calibration (the agreement between predicted probabilities and observed outcome frequencies) in addition to discrimination metrics such as sensitivity, specificity, or area under the curve; calibration is essential if a model’s output is to inform an individual stratification or treatment decision rather than simply rank patients.

Fifth, model interpretability remains limited for most connectome-based and deep-learning approaches in this literature, which constrains both clinical trust and the ability to audit a model for spurious or confounded associations. Finally, the sample sizes required for a robust, externally validated predictive model in this specific application, typically several hundred to low thousands of participants for stable connectome-based prediction, depending on effect size and model complexity, substantially exceed those of the constituent studies cited in Section 5.4.2, and would need to be an explicit design target, rather than an incidental by-product, of the nested imaging sub study described in Section 5.4.4.

Until external validation in an LAI-eligible population, prospective calibration assessment, and multi-site harmonization have been demonstrated, connectome-based response prediction should be treated in any future trial as an exploratory, hypothesis-generating stratification variable rather than a clinical decision tool.

#### 5.4.4. Integration with the Other Three Proposed Solutions and Practical Considerations

Incorporating PET and fMRI sub studies into the integrated trial design described in Section 5.3 would not require imaging in the entire trial population. A nested-cohort design, in which a subset of participants in each arm undergoes baseline (and, for PET, follow-up) imaging, is methodologically standard in psychopharmacology and would substantially limit cost and burden relative to whole-sample imaging.

PET sub studies are the more resource-intensive component, given radiotracer production, cyclotron access, and radiation exposure considerations that limit the feasible number of scans per participant; fMRI sub studies are comparatively more scalable and carry no ionizing radiation exposure, making baseline rs-fMRI a more feasible candidate for broader stratified deployment, with PET reserved for a smaller mechanistic sub cohort addressing the occupancy-stability question described in Section 5.4.1.

AI-based analysis pipelines reduce the marginal cost of both modalities by automating segmentation, motion correction, and feature extraction, but require harmonized acquisition protocols across sites to avoid scanner-related variability degrading model performance, a limitation explicitly noted in the existing ML-fMRI prediction literature [19].

Within this framework, AI-supported neuroimaging functions as a predictive and pharmacodynamic complement to, rather than a substitute for, the adherence monitoring, pharmacogenomic stratification, and pragmatic design elements already proposed: digital sensors confirm that medication was taken, pharmacogenomic stratification ensures comparable plasma exposure, pragmatic design ensures a representative population and neuroimaging-AI biomarkers provide an objective, brain-level readout of pharmacodynamic engagement and predicted response that none of the other three solutions can supply.

### 5.5. Feasibility and Sample Size Considerations

The feasibility of integrating all four solutions into a single trial protocol warrants careful consideration. Pharmacogenomic testing at baseline adds modest cost and minimal operational complexity, given the availability of commercially validated multi-gene panels with established turnaround times. Digital adherence monitoring introduces greater operational complexity, including device procurement, smartphone compatibility management, participant training, data platform maintenance, and systematic monitoring of participant experience and data quality.

Cost per participant for trial-grade digital monitoring systems is estimated at $200 to $1000, depending on platform. Pragmatic trial design reduces costs associated with restrictive exclusion screening and intensive protocol-driven procedures, potentially partially offsetting additional monitoring and genotyping costs. As discussed in Section 5.4.4, a nested neuroimaging sub study is the most resource-intensive addition, particularly for PET, and would realistically be limited to a mechanistic sub cohort rather than the full trial population; fMRI sub studies are more readily scalable and could feasibly be obtained from a larger proportion of participants at each site.

The proposed trial is a primary randomized trial, not a systematic or scoping review, and should therefore be prospectively registered on an appropriate clinical-trial registry (e.g., ClinicalTrials.gov or ISRCTN) rather than PROSPERO, which registers reviews rather than primary studies. A full protocol and a prospectively published statistical analysis plan (SAP), specifying the primary endpoint, estimand, and analysis methods in advance of unblinding, should accompany registration.

We recommend a single primary clinical endpoint: time to first psychiatric hospitalization or relapse (whichever occurs first) over 24 months, consistent with the endpoint used in the PRELAPSE trial [17] and other pragmatic LAI comparisons. The estimand should be defined explicitly as the effect of randomized assignment to LAI versus adherence-confirmed oral treatment on time to relapse, analyzed on an intention-to-treat basis; treatment switching (e.g., an oral-arm participant subsequently prescribed an LAI, or vice versa) should be handled as a protocol deviation captured in a prespecified sensitivity analysis (e.g., a per-protocol or inverse-probability-of-censoring-weighted analysis) rather than by exclusion, and reported alongside the primary intention-to-treat result.

The following sample-size illustration is presented for planning purposes only and should be revisited once the primary endpoint and expected event rates are finalized in a full protocol. Using a 24-month relapse/hospitalization rate of approximately 45% in the oral arm, informed by rates reported in prior real-world and pragmatic LAI cohorts rather than derived precisely from any single trial, and assuming a relative risk reduction with LAI treatment broadly consistent in direction with the effect reported in PRELAPSE [17], a two-sided alpha of 0.05, 80% statistical power, and an anticipated dropout rate of 20%, a minimum sample size in the order of several hundred participants per arm would likely be required for a time-to-event primary endpoint using a log-rank test; the exact figure should be calculated from the finalized event-rate assumptions at the protocol stage.

Stratified analysis by CYP2D6 metabolizer phenotype and any neuroimaging-derived subgroup (e.g., predicted pharmacodynamic responder status, Section 5.4.2) should be prespecified as secondary or exploratory analyses, with adequate enrolment within each stratum to permit meaningful comparison. Because these subgroup and secondary analyses raise multiplicity concerns, a prespecified multiplicity-control strategy (e.g., a hierarchical/gatekeeping testing procedure or a false discovery rate correction across secondary and subgroup endpoints) should be defined in the SAP, and only the single primary endpoint should be used for the trial’s confirmatory conclusion; all pharmacogenomic and neuroimaging subgroup findings should be reported and interpreted as hypothesis-generating.

## 6. Ethical and Practical Considerations

### 6.1. Digital Adherence Monitoring: Consent, Autonomy, and Surveillance

Digital adherence monitoring using ingestible sensor technology raises significant ethical considerations that must be explicitly and comprehensively addressed in any research protocol proposing its use. The most fundamental requirement is free and fully informed consent: patients must clearly understand that an electronic sensor embedded in their medication will transmit ingestion data to a digital platform accessible to their clinician and, with patient permission, to designated family members or caregivers. The consent process must transparently explain what data is collected, how it is stored, who can access it, for how long it is retained, and under what circumstances it may be disclosed or shared. Patients must be explicitly informed that participation is entirely voluntary and that refusal to use the monitoring system will not affect the quality of care they receive [21].

Patient autonomy is a particular concern in schizophrenia research, where anosognosia, impaired illness insight, is prevalent and may compromise a patient’s capacity to provide genuinely informed consent [31,34]. Research protocols must include formal mental capacity assessment at enrolment and should offer supported decision-making processes for patients with borderline capacity. The coercive potential of monitoring technology must be explicitly guarded against: digital monitoring must never be positioned as a condition of receiving antipsychotic treatment, and participants must retain the right to withdraw data sharing at any time and without consequence to their ongoing care. Furthermore, digital monitoring creates a surveillance dynamic that may be experienced as threatening, intrusive, or dehumanizing by some patients, particularly those with paranoid symptomatology. Researchers should systematically assess and report participant experience with monitoring throughout the trial period [21].

Digital equity presents a further practical and ethical concern: digital monitoring requires smartphone access, digital literacy, and reliable internet connectivity, none of which are universally available among people with schizophrenia, who experience disproportionate rates of poverty, housing instability, and social marginalization. Research protocols must include provisions for participants who do not own smartphones, must assess whether monitoring acceptability and feasibility differ systematically across socioeconomic, demographic, cultural, and linguistic groups, and must make targeted efforts to ensure representative enrolment that does not systematically disadvantage less digitally resourced participants.

### 6.2. Pharmacogenomic Research Ethics

Pharmacogenomic testing in a clinical trial context raises important considerations around genetic data privacy, incidental findings, and the potential for genetic information to influence insurance eligibility or employment decisions in some jurisdictions. Research protocols must specify whether pharmacogenomic data will be stored in biobanks, whether participants may be recontacted regarding clinically significant incidental findings, and how genetic data will be protected from unauthorized secondary use or commercial exploitation. Consent for pharmacogenomic testing should be presented and signed separately from general trial consent, thereby allowing participants to decline genetic testing whilst continuing to participate in other aspects of the study. All genetic data handling must comply with applicable national and international data protection regulations.

### 6.3. Patient and Public Involvement

No patients or members of the public were directly involved in the design or conduct of this scoping review. However, it is essential that future primary research arising from the recommendations of this review incorporates patient and public involvement (PPI) in all stages of protocol development, particularly given the significant ethical sensitivities surrounding digital monitoring technology in a population with impaired insight, and the potential for pharmacogenomic testing to generate clinically significant incidental findings. PPI representatives, including individuals with lived experience of schizophrenia, should be formally included in trial steering committees, digital monitoring acceptability assessments, and dissemination planning for any trial implementing the integrated methodology proposed here. This review indicates that the contradiction between RCT evidence of LAI-oral equivalence and real-world evidence of LAI superiority reflects four specific, addressable methodological failures rather than heterogeneity alone: the inability to measure oral-arm adherence objectively, unaddressed CYP450-driven pharmacokinetic variability, systematic exclusion of clinically representative populations from RCTs, and underuse of objective neuroimaging biomarkers. Together these failures have produced decades of contradictory findings, leaving unanswered a fundamental clinical question: when a patient is genuinely and objectively adherent to an appropriately dosed oral antipsychotic, does an LAI confer additional benefit?

None of the four proposed solutions is individually novel: digital adherence monitoring [33,50,53], pharmacogenomic testing [23,24], pragmatic trial design [17,22,32], and PET/fMRI neuroimaging [18,19,20,28] are each independently established. What is novel is their integration within a single trial design targeting this specific paradox; no existing study has combined all four within one protocol.

## 7. Priorities for AI-Supported Neuroimaging Research

Building on Section 5.4, three applications merit priority in future trial design. A head-to-head PET occupancy-stability sub study, nested within the pragmatic trial design (Section 5.3), would directly test whether LAIs’ smoother pharmacokinetics produce more stable receptor engagement than adherence-confirmed oral dosing a question no existing PET study has addressed. Baseline resting-state fMRI connectivity, analyzed with machine learning, could serve as a pre-randomization stratification variable analogous to pharmacogenomic stratification (Section 5.2), distinguishing predicted pharmacodynamic responders from non-responders and, as a secondary output, providing external validation of existing connectome-based models in an LAI-eligible population for the first time. Longer-term, multimodal AI frameworks integrating neuroimaging with pharmacogenomic and adherence data could help disentangle the relative contributions of these factors to outcome, though this extension requires the methodological safeguards discussed in Section 5.4.3. We regard PET sub studies as the most resource-intensive element of this agenda and therefore best piloted in a mechanistic sub cohort rather than treated as a precondition for the core trial (Section 5.5).

The clinical stakes extend beyond methodology. LAIs remain substantially underutilized, with penetration as low as 4% in early-phase schizophrenia despite supportive pragmatic-trial evidence [17], an underutilization partly sustained by uncertainty about the trustworthiness of the existing evidence base [6,42,45]. Definitive comparative-effectiveness evidence, generated through the integrated methodology proposed here, would either strengthen the case for LAI use or, if equivalence holds under conditions of confirmed adherence, redirect resources toward other adherence-enhancing interventions. Either outcome would meaningfully advance the evidence base for schizophrenia treatment.

Several limitations of this review should be acknowledged. As a scoping rather than a quantitative review, it is subject to potential selection bias in the literature synthesized and does not provide pooled effect estimates. The proposed integrated trial framework, while theoretically grounded, has not been empirically tested, and its feasibility in terms of cost, recruitment, regulatory requirements, and multi-site coordination requires prospective evaluation. Digital adherence monitoring has provided proof-of-concept evidence in serious mental illness populations but has not been evaluated in a definitive schizophrenia RCT, and long-term acceptability across diverse populations remains incompletely characterized [33]. Likewise, PET-based occupancy and ML-based fMRI prediction are each independently well-validated within their own studies, but neither has been combined with confirmed adherence and pharmacogenomic data within a single LAI-versus-oral trial, and the connectome-based models discussed in Section 5.4.2 require external validation in LAI-eligible populations, and the methodological safeguards outlined in Section 5.4.3, before their accuracy in this specific context can be considered established. These limitations mean the proposals presented here constitute a priority research agenda rather than established clinical practice.

## 8. Conclusions

The question of whether long-acting injectable antipsychotics are genuinely superior to oral antipsychotics for relapse prevention in schizophrenia has remained unresolved for decades, despite substantial and growing research effort. This review has demonstrated that this failure of resolution reflects systematic methodological limitations rather than an absence of research quantity: confounding by indication in observational studies, proxy-based adherence measurement that creates asymmetric information between LAI and oral arms, enrolment of unrepresentative trial populations, neglect of pharmacokinetic confounding from CYP450 genetic variability, and underuse of objective, AI-supported neuroimaging biomarkers of pharmacodynamic engagement and treatment response have collectively produced a literature that is simultaneously large and inconclusive.

Integration of objective digital adherence monitoring, with comprehensive ethical safeguards and explicit informed consent, pharmacogenomic stratification based on CYP2D6, CYP3A4, and CYP2C19 metabolizer phenotypes, pragmatic randomized trial design with real-world eligibility criteria, and AI-supported PET and fMRI neuroimaging as an objective pharmacodynamic and predictive biomarker into future comparative effectiveness research represents a necessary and feasible methodological correction. Until such evidence is generated, clinicians should interpret claims of LAI superiority derived from observational data with appropriate caution, while continuing to offer LAIs as a clinically reasonable treatment option for patients with demonstrable adherence difficulties. The research community must treat methodological innovation as a priority equivalent to drug development, recognizing that better trial design, including the principled use of neuroimaging and artificial intelligence to predict and verify treatment response, is as important as new molecules in advancing the care of people living with schizophrenia.

## Figures and Tables

**Table 3:** Summary of evidence gaps identified in part I and corresponding solutions proposed in Part II.

Section 4 Gap (Part I)	Key Finding	Consequence	Corresponding Part II Solution
Section 4.1 Confounding by indication vs. channeling bias	Confounding by indication should bias observational comparisons against LAIs; channeling bias/differential clinical monitoring instead appears to dominate real-world comparisons [12,13]	Apparent real-world LAI ‘superiority’ may substantially reflect monitoring intensity rather than the depot formulation’s pharmacology	Pragmatic trial design with blinded outcome assessment (Section 5.3)
Section 4.2 Adherence measurement failures	Oral-arm adherence is inferred from pharmacy refill data or self-report, which systematically overestimate true adherence [7,10,34]	LAI’s apparent adherence advantage may be partly or entirely artefactual, reflecting measurement asymmetry rather than pharmacology	Objective digital adherence monitoring (Section 5.1)
Section 4.3 Population generalizability	Applying typical RCT eligibility criteria to real-world registries excludes an estimated 79% of patients with schizophrenia spectrum disorders, who have worse outcomes than eligible patients [16]	RCT evidence is generated in populations systematically less ill than those for whom clinicians most need comparative-effectiveness guidance	Pragmatic trial design with broadened eligibility (Section 5.3)
Section 4.4 Pharmacokinetic/pharmacogenomic variability	CYP2D6/CYP3A4/CYP2C19 variability affects plasma concentration and adverse-effect burden; LAI administration removes first-pass/GI variability but does not eliminate CYP-mediated systemic metabolism [23,24,49]	Unmeasured genotype-driven variability may confound comparative-effectiveness estimates in both treatment arms	Pharmacogenomic stratification (Section 5.2)
Section 4.5 Unexploited neuroimaging/AI biomarkers	No existing LAI-versus-oral comparative study has incorporated PET- or fMRI-based objective pharmacodynamic or predictive markers	Comparative-effectiveness evidence lacks an objective, brain-level measure of drug engagement independent of adherence or monitoring effects	AI-supported PET/fMRI neuroimaging (Section 5.4)

*Abbreviations: LAI, long-acting injectable; RCT, randomized controlled trial; CYP, cytochrome P450; PET, positron emission tomography; fMRI, functional magnetic resonance imaging; AI, artificial intelligence.*

## Data Availability

This is a scoping review. No primary data were generated or analyzed during this study. All sources are cited in the reference list and are publicly available via PubMed or via the corresponding journal.

## Supplementary Materials

The following supporting information can be downloaded at: https://www.mdpi.com/article/10.3390/medicina62101960/s1. The following supplementary materials are submitted alongside this manuscript: Supplementary Table S1: Quality Assessment and Risk of Bias Evaluation of Included Primary Studies. Supplementary File S1 (PRISMA-ScR Checklist) [54]; and Supplementary File S2 (Full Database-Specific Search Strategies).

## Abbreviations

AI, artificial intelligence; APA, American Psychiatric Association; AMSTAR-2, A Measurement Tool to Assess systematic Reviews, version 2; CI, confidence interval; CPIC, Clinical Pharmacogenetics Implementation Consortium; CYP450, cytochrome P450; DPWG, Dutch Pharmacogenetics Working Group; FDA, Food and Drug Administration; fMRI, functional magnetic resonance imaging; HR, hazard ratio; LAI, long-acting injectable antipsychotic; MeSH, Medical Subject Headings; ML, machine learning; PET, positron emission tomography; PMID, PubMed identifier; PPI, patient and public involvement; PRECIS-2, Pragmatic-Explanatory Continuum Indicator Summary, version 2; PRISMA, Preferred Reporting Items for Systematic Reviews and Meta-Analyses; PROSPERO, International Prospective Register of Systematic Reviews; RCT, randomised controlled trial; ROBINS-I, Risk of Bias in Non-randomised Studies of Interventions; RoB 2, Cochrane Risk of Bias tool, version 2; rs-fMRI, resting-state functional magnetic resonance imaging; RR, risk ratio; SPECT, single-photon emission computed tomography.
