# Supplementary material for: The LAI–Oral Antipsychotic Efficacy–Effectiveness Paradox in Schizophrenia
Source: Medicina (Kaunas). 2026 Oct 10;62(10):1960. doi: 10.3390/medicina62101960 (PMC13706815; doi:10.3390/medicina62101960)
Supplement: Supplementary file 1 [file medicina-62-01960-s001.zip › Supplementary File S1 PRISMA-ScR_Checklist.pdf]

PRISMA-ScR Checklist

Resolving the LAI-Oral Antipsychotic Efficacy-Effectiveness Paradox in Schizophrenia: A Scoping Review (PRISMA extension for Scoping Reviews) and Proposed Precision-Medicine Framework

Based on: Tricco AC, Lillie E, Zarin W, et al. PRISMA Extension for Scoping Reviews (PRISMA-ScR): Checklist and Explanation. Ann Intern Med. 2018;169(7):467-473.

Section	Item	PRISMA-ScR Checklist Item	Reported (Y/N/NA)	Location in Manuscript
TITLE	1	Identify the report as a scoping review.	Y	Title now explicitly reads '...A Scoping Review (Reported in Accordance with PRISMA-ScR) and Proposed Precision-Medicine Framework.'
ABSTRACT	2	Structured summary including background, objectives, eligibility criteria, sources of evidence, charting methods, results, and conclusions that relate to the review questions and objectives.	Y	Abstract, p.1-2
INTRODUCTION	3	Rationale: describe the rationale in the context of what is already known; explain why the review questions/objectives lend themselves to a scoping review approach.	Y	Introduction (Section 1), p.3-4
INTRODUCTION	4	Objectives: Provide explicit statement of questions/objectives; indicate how PCC (Population/Concept/Context) elements are used.	Y	Introduction, explicit 'Aim:' statement (Section 1); Objective in Abstract
METHODS	5	Protocol and registration: Indicate whether a review protocol exists, if/where it can be accessed, and provide registration information (registry name/number).	N	-Nil
METHODS	6	Eligibility criteria: List inclusion/exclusion criteria (e.g., language, publication status) and rationale.	Y	Methods, p.5-6
METHODS	7	Information sources: Databases and other sources searched, with dates of coverage/last search.	Y	Methods: PubMed, EMBASE, PsycINFO, Cochrane Library, Jan 2000-March 2026, p.5
METHODS	8	Search: Full search strategy for at least one database, including limits, so it could be repeated.	Y	Methods lists free-text search terms, p.5; full Boolean search strings for all four databases now provided in Supplementary File 2
METHODS	9	Selection of sources of evidence: State process for selecting sources (i.e., screening and eligibility) in the review.	Y	Methods, p.5-6: dual independent screening, third-reviewer arbitration
METHODS	10	Data charting process: Describe methods of charting data, number of reviewers, calibration process, and whether done independently or in duplicate.	Y	Methods, p.5-6
METHODS	11	Data items: List and define all variables for which data were sought.	Y	Data was extracted using standerdized form including author years ,study type , quality tools , overall quality rating and key concepts

Section	Item	PRISMA-ScR Checklist Item	Reported (Y/N/NA)	Location in Manuscript
				..listed on table supplement table 1 on page 38 in manuscript.
METHODS	12	Critical appraisal of individual sources of evidence: If done, provide process and any tools used.	Y	Methods, p.6: RoB 2, ROBINS-I, AMSTAR-2; results in Supplementary Table 1
METHODS	13	Synthesis of results: Methods of handling and summarising the data charted.	Y	Methods, p.6: narrative synthesis by theme
RESULTS	14	Selection of sources of evidence: Numbers screened, assessed for eligibility, and included, ideally with a flow diagram.	Y	Fig 1 the study selection process is presented in the PRISMA 2020 flow diagram. 1553 records identified, 300 duplicates removed,1253 records screened,103 reports sought for retrieval, 100 full text reports assessed for eligibility,85 reports excluded (50 wrong population, 35 wrong study design), and 15 studies included in the review. Available on page 5 of manuscript.
RESULTS	15	Characteristics of sources of evidence: Characteristics of included sources (e.g., study design, population, context).	Y	Summary of Evidence on LAI Versus Oral Antipsychotics by Study Design, Key Findings, Limitations, and Methodological Gaps Addressed by the Four Proposed Solutions on page 36 of the manuscript
RESULTS	16	Critical appraisal within sources of evidence: Present results of critical appraisal, if done.	Y	methodological quality of included sources of evidence was assessed using validated appraisal tools (RoB 2, ROBINS-I, AMSTAR 2). The quality assessment findings are summarized in Supplementary Table 1 and described in Section 2.1 of the manuscript
RESULTS	17	Results of individual sources of evidence: Present results for each included source relevant to the review questions/objectives.	Y	Section 3.3, Table 1, and narrative synthesis throughout Sections 3-5
RESULTS	18	Synthesis of results: Summarise/present the charting results as they relate to the review questions/objectives.	Y	Sections 3-7

Section	Item	PRISMA-ScR Checklist Item	Reported (Y/N/NA)	Location in Manuscript
DISCUSSION	19	Summary of evidence: Summarise main results, relate to review questions/objectives, and consider relevance to key groups.	Y	Discussion (Section 7)
DISCUSSION	20	Limitations: Discuss limitations of the scoping review process.	Y	Explicit standalone limitations paragraph at the end of Discussion (Section 7)
DISCUSSION	21	Conclusions: Provide a general interpretation of results, with consideration of implications for future research and practice.	Y	Conclusions (abstract) and Discussion, Section 7
FUNDING	22	Funding: Sources of funding and role of funders.	N	nill

Notes:. Item 5 is honestly reported as 'N': this scoping review was not prospectively registered (PROSPERO does not accept scoping-review registrations). Item 14's PRISMA flow-diagram record counts and Item 14/Section 2.1's Cohen's kappa value remain placeholders pending the authors' final search and screening records and must be completed before submission.
