# Supplementary material for: The LAI–Oral Antipsychotic Efficacy–Effectiveness Paradox in Schizophrenia
Source: Medicina (Kaunas). 2026 Oct 10;62(10):1960. doi: 10.3390/medicina62101960 (PMC13706815; doi:10.3390/medicina62101960)
Supplement: Supplementary file 1 [file medicina-62-01960-s001.zip › Supplementary File S2 Search_Strategies.pdf]

Supplementary File S2

Full Database-Specific Search Strategies

The conceptual search string common to all databases combined long-acting injectable antipsychotic terms, oral antipsychotic terms, and schizophrenia terms, further filtered by the domain-specific terms listed in the Methods section (Section 2) of the manuscript. Database-specific syntax, controlled-vocabulary (subject heading) terms, and field tags are given below for each of the four databases searched.

PubMed (MEDLINE)

Date range searched: January 2000 - March 2026

```
("long-acting injectable antipsychotics"[tiab] OR "long-acting injectable
antipsychotic"[tiab] OR "LAI antipsychotic*"[tiab] OR "depot antipsychotic*"[tiab]
OR "Delayed-Action Preparations"[Mesh]) AND ("oral antipsychotic*"[tiab] OR
"Antipsychotic Agents/administration and dosage"[Mesh]) AND ("schizophrenia"[tiab]
OR "Schizophrenia"[Mesh]) AND ("medication adherence"[tiab] OR "Medication
Adherence"[Mesh] OR "treatment adherence"[tiab] OR "confounding by
indication"[tiab] OR "pharmacogenomics"[tiab] OR "Pharmacogenomic Testing"[Mesh] OR
"CYP2D6"[tiab] OR "Cytochrome P-450 CYP2D6"[Mesh] OR "digital medicine"[tiab] OR
"digital health"[tiab] OR "pragmatic trial*"[tiab] OR "Pragmatic Clinical
Trial"[Publication Type] OR "positron emission tomography"[tiab] OR "Positron-
Emission Tomography"[Mesh] OR "functional magnetic resonance imaging"[tiab] OR
"Magnetic Resonance Imaging"[Mesh] OR "neuroimaging"[tiab] OR "Neuroimaging"[Mesh]
OR "machine learning"[tiab] OR "Machine Learning"[Mesh] OR "artificial
intelligence"[tiab] OR "Artificial Intelligence"[Mesh])
Filters: 2000/01/01-2026/03/31; Humans
```

EMBASE (Ovid/Elsevier interface)

Date range searched: January 2000 - March 2026

```
('long acting injectable antipsychotics':ti,ab OR 'long acting injectable
antipsychotic':ti,ab OR 'lai antipsychotic*':ti,ab OR 'depot antipsychotic*':ti,ab
OR 'depot antipsychotic agent'/exp) AND ('oral antipsychotic*':ti,ab OR
'antipsychotic agent'/exp) AND ('schizophrenia':ti,ab OR 'schizophrenia'/exp) AND
('medication adherence':ti,ab OR 'patient compliance'/exp OR 'treatment
adherence':ti,ab OR 'confounding by indication':ti,ab OR 'confounding bias'/exp OR
'pharmacogenomics':ti,ab OR 'pharmacogenomics'/exp OR 'cyp2d6':ti,ab OR 'cytochrome
p450 2d6'/exp OR 'digital medicine':ti,ab OR 'digital health':ti,ab OR 'pragmatic
trial*':ti,ab OR 'pragmatic clinical trial'/exp OR 'positron emission
tomography':ti,ab OR 'positron emission tomography'/exp OR 'functional magnetic
resonance imaging':ti,ab OR 'functional magnetic resonance imaging'/exp OR
'neuroimaging':ti,ab OR 'neuroimaging'/exp OR 'machine learning':ti,ab OR 'machine
learning'/exp OR 'artificial intelligence':ti,ab OR 'artificial intelligence'/exp)
Limits: 2000-2026; human; article, article in press, review
```

PsycINFO (EBSCOhost/Ovid interface)

Date range searched: January 2000 - March 2026

```
(TI,AB("long-acting injectable antipsychotics" OR "long-acting injectable
antipsychotic" OR "LAI antipsychotic*" OR "depot antipsychotic*") OR DE
"Neuroleptic Drugs") AND (TI,AB("oral antipsychotic*") OR DE "Antipsychotic Drugs")
AND (TI,AB("schizophrenia") OR DE "Schizophrenia") AND (TI,AB("medication
adherence" OR "treatment adherence" OR "confounding by indication" OR
"pharmacogenomics" OR "CYP2D6" OR "digital medicine" OR "digital health" OR
"pragmatic trial*" OR "positron emission tomography" OR "functional magnetic
resonance imaging" OR "neuroimaging" OR "machine learning" OR "artificial
intelligence") OR DE "Treatment Compliance" OR DE "Pharmacogenetics" OR DE
"Positron Emission Tomography" OR DE "Neuroimaging" OR DE "Machine Learning" OR DE
"Artificial Intelligence")
Limits: Publication Year 2000-2026; Human
```

Cochrane Library (CENTRAL, Cochrane Reviews)

Date range searched: January 2000 - March 2026

```
("long-acting injectable antipsychotics" OR "long-acting injectable antipsychotic"
OR "LAI antipsychotic*" OR "depot antipsychotic*"):ti,ab,kw AND ("oral
antipsychotic*"):ti,ab,kw AND ("schizophrenia"):ti,ab,kw AND ("medication
adherence" OR "treatment adherence" OR "confounding by indication" OR
"pharmacogenomics" OR "CYP2D6" OR "digital medicine" OR "digital health" OR
"pragmatic trial*" OR "positron emission tomography" OR "functional magnetic
resonance imaging" OR "neuroimaging" OR "machine learning" OR "artificial
intelligence"):ti,ab,kw
Publication dates: Jan 2000 to Mar 2026; Trials and Reviews
```

Note: Search strategies are reported as executed (or as adapted to each platform's syntax) to allow replication, per PRISMA-ScR Item 8. Truncation (*) and phrase-searching conventions follow each platform's standard syntax. Reference lists of all included studies and relevant prior reviews were additionally hand-searched for eligible records not captured by the electronic search.
