# Supplementary material for: The LAI–Oral Antipsychotic Efficacy–Effectiveness Paradox in Schizophrenia
Source: Medicina (Kaunas). 2026 Oct 10;62(10):1960. doi: 10.3390/medicina62101960 (PMC13706815; doi:10.3390/medicina62101960)
Supplement: Supplementary file 1 [file medicina-62-01960-s001.zip › Supplementary Table S1.pdf]

## Supplementary Table S1. Quality Assessment of Key Primary Studies Included in This Review

RCTs assessed using Cochrane Risk of Bias tool version 2 (RoB 2). Observational studies assessed using ROBINS-I. Systematic reviews and meta-analyses assessed using AMSTAR-2.

| Author (Year) | Study Type | Quality Tool | Overall Quality Rating | Key Concern |
|---|---|---|---|---|
| Kishimoto et al. 2014 [10] | Systematic review and meta-analysis of RCTs | AMSTAR-2 | Moderate (high confidence) | Significant heterogeneity across included RCTs; variable follow-up durations. |
| Schneider-Thoma et al. 2022 [11] | Network meta-analysis of RCTs | AMSTAR-2 | High (high confidence) | Network inconsistency in some comparisons; mixed active and placebo comparators. |
| Kane et al. 2020 [32] | Cluster-randomised pragmatic RCT | RoB 2 | Low-to-moderate | Cluster randomisation introduces contamination risk; intention-to-treat analysis may dilute effect. |
| Tiihonen et al. 2017 [9] | Nationwide cohort study | ROBINS-I | Moderate | Confounding by indication despite registry adjustment; proxy adherence data; co-intervention confounding. |
| Taipale et al. 2022 [37] | Cohort applying RCT criteria to registry data | ROBINS-I | Low | Large representative registries; well-defined eligibility criteria; minimal missing data. |
| Lin et al. 2021 [7] | Systematic review and meta-analysis of real-world studies | AMSTAR-2 | Moderate | All included studies observational; adherence by proxy; high heterogeneity across database sources. |
| Kishimoto et al. 2013 [34] | Systematic review of mirror-image studies | AMSTAR-2 | Moderate | Pre-post design subject to regression to the mean; naturalistically selected populations; no control group. |
| Taipale et al. 2024 [35] | Network meta-analysis combining RCT and real-world data | AMSTAR-2 | High (high confidence) | Methodologically innovative; some network inconsistency in combined analyses. |
| Bousman et al. 2024 [16] | Systematic review of pharmacogenomic testing | AMSTAR-2 | Moderate-to-high | Heterogeneous pharmacogenomic testing protocols; limited antipsychotic-specific subgroup data. |
| Niarchou et al. 2022 [42] | Two-site provider-randomised feasibility trial | RoB 2 | Low-to-moderate | Small sample (n=46); short duration (90 days); feasibility outcome only; no clinical effectiveness endpoint. |

AMSTAR-2, A MeaSurement Tool to Assess systematic Reviews, version 2; ROBINS-I, Risk of Bias in Non-randomised Studies of Interventions; RoB 2, Cochrane Risk of Bias tool, version 2.
